# Supplementary material for: Rooting ability of rice seedlings increases with higher soluble sugar content from exposure to light
Source: PLoS One. 2020 Oct 20;15(10):e0241060. doi: 10.1371/journal.pone.0241060 (PMC7575106; doi:10.1371/journal.pone.0241060)
Supplement: Attachment — Submitted filename: renamed_a10f8.pdf [file pone.0241060.s001.pdf]

Dear Editor,

Thank you very much for giving us the opportunity to restate our point of view.

This study aimed to explore the relationship between endogenous sugars and rooting ability of rice seedlings, and to find out some measures that can be directly used to improve the rooting ability of rice seedlings for mechanical transplanting. We achieved the regulation of endogenous sugar content by controlling the time of illumination, and proved that it has a very close relationship with rooting ability, and this find can be used to guide practical application.

We have already delivered this article to this journal previously. However, as illumination time was taken as the control variable in our experiment, and sampling and analysis tests occurred at different time points, so compared with the common experiments design, that is, simultaneous implementation of experimental processing and then analysis and testing, readers may not be easy to understand our experiment design. On the other hand, our expression was not concise enough and easy to understand, it may confuse the previous reviewers. All these may lead to the rejection of our last submission. This time, we have made many improvements in the title, introduction, experimental methods, result elaboration, discussion and conclusion. We think it's a new manuscript, so we resubmitted it and hope to publish it in this journal.

Specifically, the main improvements are as follow.

1. We 've revised the title of the article from "Rapidly enhanced rooting ability associated with an increased shoot soluble sugar content of rice seedlings under light" to "Rooting ability of rice seedlings increases with higher soluble sugar content from exposure to light", which is more concise to reflect the research content of this paper and easier to understand.
2. We 've revised the main conclusion from "soluble sugar content before rooting played a key role in determining the rooting ability of the rice seedlings" to "compared with nitrate-nitrogen content, soluble sugar content was more dominant in affecting rooting ability", which can get full support from our experimental results as well as other studies.
3. We 've made corresponding supplements and modifications in results section and discussion section. We 've rewritten the interpretation on the results of the correlation analysis and supplemented partial correlation analysis. We've almost completely rewritten the discussion section to fully elucidate the relationship between soluble sugars, nitrate nitrogen and root ability, and their relative influence on root ability.
4. We've added the research progress on the relationship between sugar and root formation in the introduction section (Paragraph 3).
5. We've rewritten the last paragraph of the introduction section. This study aimed to explore the relationship between endogenous sugars and rooting ability of rice seedlings, so as to provide guidance for finding out some measures that can be directly used to increase the rooting ability of rice seedlings for mechanical transplanting.

6. We’ve added a new part (2.2 Treatments and their implementation) to the Materials and Methods section, which we believe to make our methods easier to understand. We also have rewritten the statistical analysis section.
7. We’ve added a concise conclusion section to summarize the main conclusions of this study and its application value.

We try our best to improve our article, but if there are still problems, please don't hesitate to tell us, we will improve further.

A response to previous comments is attached bellow.

Thank you very much for your attention to our paper.

Sincerely yours

Wanlai Zhou

Response to Reviewer 1 Comments

Point 1: The manuscript presentments interesting data and could be considered for publication after a major revision. The study presents the results of original research, and no data have been published elsewhere. Nevertheless, experiments, statistics, and other analyses are performed to a low technical standard and are not described in sufficient detail.

Response 1: We have made many improvements in the title, introduction, experimental methods, result elaboration, discussion and conclusion.

Point 2: Conclusions are not presented in an appropriate fashion and are not supported by the data in a convincing manner. Consequently, the article is not presented in an intelligible fashion.

Response 2: We 've revised the main conclusion from “soluble sugar content before rooting played a key role in determining the rooting ability of the rice seedlings” to “compared with nitrate-nitrogen content, soluble sugar content was more dominant in affecting rooting ability”, which can get full support from our experimental results as well as other studies. Accordingly, we 've made supplements and modifications in results section and discussion section. We 've rewritten the interpretation on the results of the correlation analysis and supplemented partial correlation analysis. We've almost completely rewritten the discussion section.

Point 3: To the MS is written in standard English, there are some technical errors that must be addressed by the authors.

Response 3: Before we submit this paper, we have adopted a professional revision service (https://www.letpub.com.cn/). We are willing to make further amendments after pointing out the specific mistakes.

Point 4: For reader’s information, all rough data and statistical analyses must be attached as Supplementary Information.

Response 4: We believe that the charts and Tables in the article have provided enough data information, and we can supplement all the original data if required by the journal.

Response to Reviewer 2 Comments

Point 1: The study theme is interesting but after peer reviewing, I found the following shortfalls in the overall structure of MS that must be considered according to the standards of this great journal, The abstract section just focused on the soluble sugar contents in rice seedlings and not a single other parameter is mentioned to support the study objectives, results or conclusion

Response 1: In this study, we attempted to obtain rice seedlings different in soluble sugar content but roughly uniform in other aspects by controlling the illumination time and we succeeded. The soluble sugar content of the rice seedlings before rooting increased rapidly while few significant changes in the other physiological indices were detected. With the light exposure time increasing from 3 hours to 12 hours, the rooting ability increased significantly. Through correlation analysis and regression analysis, we believe that the soluble sugar content was more dominant in affecting rooting ability compared with nitrate-nitrogen content. **We pointed out the fact that few significant changes in the other physiological indices were detected in the abstract, which we think is clear and there is no more useful information on these physiological indices to be elaborated.**

Point 2: Introduction section doesn't end with proper objectives of the study or highlight the study objectives.

Response 2: We rewrote the last paragraph of the introduction section. This study aimed to explore the relationship between endogenous sugars and rooting ability of rice seedlings, so as to provide guidance for finding out some measures that can be directly used to increase the rooting ability of rice seedlings for mechanical transplanting. We realized the regulation of endogenous sugar content by controlling the time of illumination, and proved that it has a very close relationship with rooting ability, and this find can be used to guide practical application.

Point 3: The material and methods section is not clear regarding growth, 'rooting ability' measurement and the root-cut rice seedlings planting method. Statistical analysis section should be brief and concise.

Response 3: In our experiment, illumination time was taken as the control variable, and sampling and analysis tests occurred at different time points. Therefore, compared with the common experiments design, that is, simultaneous implementation of experimental processing and then analysis and testing, readers may not be easy to understand our experiment design. Therefore, we added a new part (2.2 Treatments and their implementation) to the materials and methods section, which we believe to make our methods easier to understand. We also have rewritten the statistical analysis section.

Point 4: The study results are confusing because morphological parameters were significantly affected by treatments (3 to 12 h) while physiological parameters were non-significant as mentioned at L 231. Lines 189 to 193 reporting that just light exposure from 3 to 12 h duration FW, DW and total root lengths increased by 91.7, 120 and 60.6 % respectively and after 12 to 24 h the treatment effect was usually non-significant which is beyond my imaginations.

Response 4: As we said above, in our experiment, illumination time was taken as the control variable. Specifically, after different duration time of light (0, 3, 6, 9, 12, 24 hours), rice seedlings were taken out for rooting ability test and physiological analysis. Obviously, it can be expected that the longer the light time, the larger the plant height. In our experiment, the plant height of rice seedlings conformed to this trend, but the difference in plant height among rice seedlings after different illumination (0, 3, 6, 9, 12, 24 hours) did not reach a significant level. In fact, there was no significant change in physiological indexes of rice seedling under such light except the soluble sugar content. At the same time, there were significant differences in rooting ability of seedlings after different periods of light. Therefore, we can attribute the difference in rooting ability to the change of soluble sugar content of rice seedlings.

Point 5: There are not enough parameters to prove the study objectives.

Response 5: This study aimed to explore the relationship between endogenous sugars and rooting ability of rice seedlings, and to find out some measures that can be directly used to improve the rooting ability of rice seedlings for mechanical transplanting. We achieved the regulation of endogenous sugar content by controlling the time of illumination, and proved that it has a very close relationship with rooting ability, and this find can be used to guide practical application.

Point 7: MS needs significant improvement regarding English language

Response 7: Before we submit this paper, we have adopted a professional revision service (https://www.letpub.com.cn/). We are willing to make further amendments after pointing out the specific mistakes.
