# Supplementary material for: Rooting ability of rice seedlings increases with higher soluble sugar content from exposure to light
Source: PLoS One. 2020 Oct 20;15(10):e0241060. doi: 10.1371/journal.pone.0241060 (PMC7575106; doi:10.1371/journal.pone.0241060)
Supplement: Attachment — Submitted filename: Response to Reviewers Comments.docx [file pone.0241060.s002.docx]

Dear editor,

Thank you for giving us the opportunity to submit a revised draft of the manuscript “Rooting ability of rice seedlings increases with higher soluble sugar content from exposure to light” for publication in PLOS ONE. We appreciate the time and effort that you and the reviewers dedicated to providing feedback on our manuscript and are grateful for the insightful comments on and valuable improvements to our paper.

We have incorporated most of the suggestions made by the reviewers and adjusted the format of the manuscript according to the format guide of PLOS ONE. Those changes are highlighted within the manuscript. Please see below, in red, for a point-by-point response to the reviewers’comments and concerns.

Your sincerely,

Wanlai Zhou

Response to Reviewer 1 Comments
Point 1: In its present form, I feel authors fails to highlight the novelty of this study. During active growth phase most of the energy coming through respiration is utilized for growth process for example rooting in the early seedling establishment phase. This energy is certainly coming by the breakdown of simple sugars. Thus, it is very obvious to get a strong and positive correlation between rooting ability and endogenous sugars. Moreover, more light would increase amount of photo-assimilates until a saturation point. On the other hand, rooting ability is a complex trait which is tightly regulated by genetic background and genotype x environment interaction. Authors should discuss in more details, how increase in assimilates partitioned towards root. As roots could be main sink during early growth, traits such as leaf area, photosynthetic ability and root architecture may be crucial to determine overall rooting ability
Response 1: Thank you very much for your professional and kind suggestions. We agree with your assessment, your suggestions have opened our mind and we have reorganized the discussion section of our article to highlight the novelty of our study. Based on the experimental data we got, we adjusted our discussion points to “the substances determining rooting ability in rice seedlings under light changed with the duration of light” and “soluble sugar content was more dominant in determining rooting ability compared with nitrate-nitrogen content”. Indeed, it is very obvious to get a strong and positive correlation between rooting ability and endogenous sugars. However, our results showed that it’s not so simple. Rooting ability is a complex trait, and our article demonstrated the influence of light on rooting ability and the complexity of the effect of light duration on rooting ability, and compared the relative importance of the effects of endogenous soluble sugar and nitrate nitrogen. Based on this, we put forward some suggestions that can guide the rice mechanical transplanting of rice.

Response to Reviewer 2 Comments
Point 1: The research experiment was well executed with proper statistical support. The results were well presented and discussed with proper references. The comments of earlier reviewers were properly addressed and the same reflected in the MS. With this the MS entitled "Rooting ability of rice seedlings increases with higher soluble sugar content from exposure to light" can accepted for publication.
Response 1: Thank you for your comments.

Response to Reviewer 3 Comments
Point 1: Overall this is a well written manuscript. However a minor improvement is required in the introduction section. Exposure to light is the main treatment of this manuscript but in the last paragraphs of introduction section only 1-2 general sentences have been added about the variation of soluble sugars in response to light exposure. I suggest add more specific literature in last paragraphs of the introduction section to justify the of selection of this treatment.
Response 1: Thank you very much for your professional and kind suggestions. We have made corresponding modifications according to your suggestions. In the introduction, we use a separate paragraph (line 74-83) to introduce the fluctuation of soluble sugar content in plants under light with detailed data.
